# Rethinking status 1A criteria in pediatric cardiac transplantation: A case for the prioritization of patients with single ventricle anatomy supported by ventricular assist devices

**DOI:** 10.3389/fped.2023.1057903

**Published:** 2023-02-24

**Authors:** Anna E. Berry, David W. Bearl

**Affiliations:** ^1^ Internal Medicine-Pediatrics Residency Program, Monroe Carell Jr. Children’s Hospital and Vanderbilt University Hospital, Vanderbilt University Medical Center, Nashville, TN, United States; ^2^ Division of Pediatric Cardiology, Department of Pediatrics, Monroe Carell Jr. Children's Hospital, Vanderbilt University Medical Center, Nashville, TN, United States

**Keywords:** pediatric cardiac transplantation, congential heart disease, single ventricle, transplant allocation, pediatric heart failure, ventricular assist device

## Abstract

Over the past 2 years advancements in the techniques and technology of pediatric heart transplantation have exponentially increased. However, even as the number of pediatric donor hearts has grown, demand for this limited resource continues to far outpace supply. Thus, lifesaving support in the form of ventricular assist devices (VAD) has become increasingly utilized in bridging pediatric patients to cardiac transplant. In the current pediatric heart transplant listing criteria, adopted by the United Network for Organ Sharing (UNOS) in 2016, all pediatric patients with a VAD are granted 1A status and assigned top transplant priority regardless of their underlying pathology. However, should this be the case? We suggest that the presence of a VAD alone may not be sufficient for status 1A listing. In doing so, we specifically highlight the heightened acuity, resource utilization, risk profile, and diminished outcomes in patients with single ventricle physiology supported with VAD as compared to patients with structurally normal hearts who would both be listed under 1A status. Given this, from a distributive justice perspective, we further suggest that the lack of granularity in current pediatric cardiac transplant listing categories may inadvertently lead to an inequitable distribution of donor organs and hospital resources especially as it pertains to those with single ventricle anatomy on VAD support. We propose revisiting the current listing priorities in light of improved techniques, technology, and recent data to mitigate this phenomenon. By doing this, pediatric patients with single ventricle disease might be more equitably stratified while awaiting heart transplant.

## Introduction

Over the past decade, technological advances in medicine have progressed at a rapid rate. Both in the adult and pediatric population, this is exceedingly apparent in the realm of heart failure therapies and mechanical circulatory support (MCS) devices. Innovative advances in heart failure therapies such as transplantation, percutaneous interventions, and MCS have allowed for the mitigation of heart failure and its sequalae to foster improved outcomes for this population (1, 2). Specifically, the introduction of ventricular assist devices (VAD) as a bridge to cardiac transplantation has significantly influenced the trajectory of pediatric patients with heart failure of various etiologies. However, as technology has continued to progress, these advances have shown to benefit some populations more than others. This is especially true for patients with single ventricle (SV) cardiac disease who are reliant on VAD support while awaiting transplant. Given this, we suggest that there is a need for further granularity in listing criteria as it relates to these patients. By accomplishing this, it might be possible to further ensure the just and equitable allocation of pediatric donor hearts while potentially further reducing waitlist mortality for these complex patients.

## Mechanical circulatory support: a changing landscape

Following their introduction in adults with cardiac compromise, MCS devices—such as the VAD—began to play an integral role in the management of pediatric heart failure in the 1990s (3). Given that the demand for pediatric donor hearts continues to outpace supply, these devices have become increasingly utilized as a bridge to cardiac transplantation over the past several years and have been effective in reducing waitlist mortality (1, 4). Along with FDA approval for two devices in pediatrics, VADs have become a widely utilized form of support among pediatric patients awaiting cardiac transplant throughout the past decade.

Though there has been significant improvement in waitlist mortality, VADs are not without complications. The earliest VADs used in pediatrics were associated with substantial morbidity and mortality due to pump failure, thrombosis, stroke, and infection. With improvements in device technology and patient management over the past several years, morbidity from MCS devices has dropped significantly. For example, use of the Berlin Heart EXCOR (Berlin Heart GmbH, Berlin, Germany) with improved anticoagulation mangement was found to have a ∼90% survival to transplant rate along with reduced incidence of cerebral thrombosis. Also recently introduced, the HeartMate 3 (Abbott Labs, Lake Bluff, IL, USA) device has a greatly improved complication profile including near negligible risk of stroke (5). Using the adult devices, some pediatric patients are able to avoid prolonged hospitalization as they await transplant in the outpatient setting after VAD placement (3). Overall, the relatively rapid evolution of VAD technology, patient selection, and clinical management has markedly improved outcomes for pediatric heart failure patients awaiting transplant.

## Differences matter: considerations for VAD implantation in SV heart failure

Though the introduction of VADs as a bridge to pediatric heart transplantation has shown promising progress, the benefits of VAD insertion are not uniformly distributed across all pediatric heart failure patients. The wide breadth of anatomic and physiologic differences in the pediatric heart failure population results in varying outcomes for patients placed on a VAD while awaiting transplant. Most notably, patients with congenital heart disease (CHD) often have structural and physiologic abnormalities that underlie their heart failure and complicate the course of VAD bridge to transplant as compared to patients with a structurally normal heart (6, 7).

This distinction is especially evident in the SV population, where patients often undergo surgical palliation prior to transplant and have residual complex structural cardiac lesions. Though VAD implantation has shown to be effective in bridging patients with SV CHD to transplant, physiologic and anatomical implications of these residual lesions lend to increased morbidity and require additional considerations when implementing VAD support (8). Due to anatomic variability, additional cross-sectional or 3D imaging has been used to help with surgical planning (9). Special physiologic considerations for the SV population include the comparatively increased burden of collateralization, pulmonary vascular remodeling, arrhythmias, and atrioventricular valve dysfunction (8, 10, 11). An additional concern is the higher rates of HLA sensitization for patients with CHD (12). This is suspected to be heightened in SV disease as a consequence of repeated allograft exposures and thought to be compounded by the use of whole-blood in those requiring VAD support (13). For those in interstage periods, physiologic complications potentially necessitate subsequent interventions such as Glenn takedown in order to optimize VAD support (14).

Further contributing to their complexity, many CHD patients with acute heart failure (AHF) have concomitant extracardiac comorbidities. Acutely, patients tend to suffer from respiratory and renal failure, whereas gastrointestinal diagnoses make up the majority of chronic extracardiac comorbidities. Around half of these patients are technologically dependent (4). Furthermore, elevated risk of nosocomial infections, deconditioning, and diminished cognitive outcomes has also been demonstrated for patients with CHD (15–17). These additional considerations highlight the added complexity and morbidity of CHD and SV physiology in implementing MCS.

Importantly, patients with SV physiology also suffer higher mortality while on VAD support awaiting transplant. Broadly speaking, a diagnosis of CHD has been associated with increased risk of mortality and decreased transplantation rates after 6 months with a VAD as compared to heart failure with the absence of a CHD diagnosis (18). Patients with single ventricle CHD are even more at risk for poor outcomes because they tend to be younger, smaller, and have other medical comorbidities such as impaired renal function as compared to their counterparts with biventricular CHD. Though mortality was not shown to differ between SV-CHD and non-SV CHD when adjusted for smaller size, acuity, and medical complexity, these factors are certainly at play in the practical care of these patients and should be accounted for when considering the relative risk of prolonged dependence on VAD support as a bridge to transplant (18). In another multicenter study, a diagnosis of SV heart disease was found to be associated with increased risk of mortality in patients with AHF (4). Additional investigations have revealed up to a two-fold increase in the risk of death for SV patients with VAD as compared to those with normal cardiac anatomy (19, 20). Overall, outcomes in patients with SV CHD are consistently poorer than those with structurally normal hearts. Given this, prioritizing cardiac transplantation in this high-risk population may be prudent.

The increased morbidity and mortality that SV patients experience also has significant impacts on resource utilization. Though the implantation of a VAD previously committed pediatric patients with heart failure to an inpatient stay, newer devices with improved safety profiles have begun to change this practice. In fact, the number of pediatric patients discharged with VAD support has increased over time (4, 21). Given their anatomical complexity and propensity for complications with VAD as previously discussed, patients with SV disease often are unable to return home safely and frequently have prolonged inpatient hospital stays (22). The increased resource utilization associated with these hospitalizations are further magnified by patient complexity. As previously discussed, patients with SV disease have a high burden of technology dependence, acute and chronic extracardiac comorbidities, and complications that lend to their morbidity and resource utilization. Importantly, this also exacerbates the financial burden on families in this already resource-intensive population (4, 15, 23). To mitigate this, it is likely beneficial to minimize time spent on the transplant waitlist for patients with SV heart disease on VAD support.

## Discussion: implications for transplantation waitlist status

Transplantation medicine is unique in that organ allocation policies play an integral role alongside advances in diagnostic techniques and therapeutic innovation. The field of transplant medicine is fraught with ethical dilemmas as demand for donor organs continues to exceed supply. To ensure the just allocation of this scarce resource, it is critical that policies regarding transplant waitlist stratification continue to evolve with the changing landscape of medical therapeutics. This is particularly true in pediatric heart failure, where—as previously mentioned—medical technology and therapeutic techniques continue to evolve rapidly with significant implications on outcomes. Much is at stake as we consider the way in which pediatric patients awaiting heart transplant should be prioritized. Stratification systems that are constructed from up-to-date evidence both optimize the survivability of pediatric heart failure and ensure the just allocation of pediatric donor hearts.

In partnership with the Organ Procurement & Transplantation Network (OPTN), the United Network for Organ Sharing (UNOS) seeks to justly and fairly allocate organs to those in need of transplantation. The tiered system implemented by UNOS seeks to prioritize patients with high acuity that would most benefit from timely organ transplantation. This is primarily in keeping with a concept of distributive justice in which limited resources are allocated to those who are most in need of them (24). Thus, the primary goal of waitlist stratification in pediatric heart failure is to reduce waitlist mortality by prioritizing transplant in high acuity patients who meet certain criteria that makes transplantation a more urgent matter.

To ensure the continued ethical allocation of donor organs as medical research and technology advances, UNOS criteria is revised periodically. In the case of pediatric heart transplantation, the most recent update occurred in 2016 and included four tiers for organ allocation stratification. In this rendition, the highest transplant priority (status 1A) is assigned to the following criteria: admitted to the hospital and requiring continuous mechanical ventilation, intra-aortic balloon pump, presence of a ductal dependent lesion maintained with prostaglandins or stent, CHD requiring multiple inotropic agents for hemodynamic support. Status 1A is also assigned to patients who may or may not be admitted to the hospital that require assistance of MCS including a VAD or by exception as determined by a physician (25). Thus, all patients with a VAD placement, regardless of cardiac diagnosis or inpatient status, can be listed highest priority.

However, as previously described, the population of patients with a VAD is comprised of a large range of acuity and pathology. This is especially evident in the prior description of patients with SV heart disease who have an elevated risk of complications and mortality while awaiting transplant. Though early VADs were associated with complications experienced across etiologies of pediatric heart failure, VADs introduced more recently have greatly improved support for certain subsets of patients, namely dilated cardiomyopathy, compared to others. Thus, the chasm has widened between these high-risk patients and those with normal cardiac structure where reduced VAD complications and outpatient management may be possible. Given this, the listing criteria should be reconsidered to better address discrepancies in waitlist and post-transplant outcomes among different VAD populations.

To reflect the aforementioned differences, increasing granularity pertaining to patients supported by VAD as a bridge to transplant is a reasonable step forward. Since transplant listing criteria is, at least in part, designed to reduce waitlist mortality, it would follow that a higher priority should be available for those with SV disease supported by VAD compared to those with structurally normal hearts. In adult transplantation, this distinction is appreciated. For example, persons with biventricular anatomy who must remain inpatient on VAD support are listed as status 2, whereas those who might be dischargeable with LVAD in place might be initially listed as status 3 with transition to status 4 after 30 days. However, relatively higher prioritization is appreciated for those with SV disease dependent on VAD or total artificial heart support as they are listed as status 2 (25). Given this, adult cardiac transplantation listing criteria may provide a model of stratification by which increased granularity and prioritization of patients with SV physiology—thereby accounting for their higher acuity and risk of mortality—might be accomplished.

If this strategy is implemented in pediatric patients, waitlist mortality, time to transplantation, and post-transplantation outcomes for biventricular patients with VAD would need to be closely monitored for unintended negative outcomes. For example, it is conceivable that prioritizing transplantation for patients with SV disease supported by a VAD could lead to longer wait times for their biventricular counterparts. Though most VAD-related complications decrease with time from implementation, prolonging the time dependent on a VAD while awaiting transplant could increase the cumulative risk of VAD-related morbidity (26–29). While prioritization of SV patients supported by VAD could serve to mitigate their early mortality, unintended negative ramifications for other patient populations awaiting cardiac transplant should be considered (19). Benefits to the SV population must then be carefully weighed against risks, if any, incurred by the remainder of pediatric patients awaiting cardiac transplantation.

## Conclusion

Pediatric heart failure is a complex field in which medicine, policy, and ethics are intimately intertwined. For pediatric patients awaiting cardiac transplant, UNOS listing guidelines attempt to give priority based on medical urgency in light of a limited resource. John Rawls points to this arrangement as a form of justice (25). Listing criteria must continually evolve with advances in medical technology and research to accomplish this important goal. As highlighted, recent advances in pediatric VAD have been remarkably successful in mitigating VAD-associated complications in patients awaiting transplant. However, this progress has inadvertently led to relative disparities for patients with SV heart disease who may not experience the full breadth of benefits from advances in VAD technology. For this reason, similar to changes in adult listing criteria a decade ago, revision of current pediatric listing criteria to allow for a more granular stratification and prioritization of those with SV heart disease based on their medical urgency should be employed.

## Data Availability

The original contributions presented in the study are included in the article/Supplementary Material, further inquiries can be directed to the corresponding author.
